# iTRAQ-Based Proteomic Analysis of Watermelon Fruits in Response to *Cucumber green mottle mosaic virus* Infection

**DOI:** 10.3390/ijms21072541

**Published:** 2020-04-06

**Authors:** Xiaodong Li, Xinyue Bi, Mengnan An, Zihao Xia, Yuanhua Wu

**Affiliations:** 1 College of Plant Protection, Shenyang Agricultural University, No.120 Dongling Road, Shenyang 110866, China; lxdong1221@126.com (X.L.); 2018200126@stu.syau.edu.cn (X.B.); anmengnan1984@163.com (M.A.); 2 General Station of Forest and Grassland Pest and Diseases Control, National Forestry and Grassland Administration/Key Laboratory of State Forestry and Grassland Administration on Forest Pest Monitoring and Warning, No.58 Huanghe North Street, Shenyang 110034, China

**Keywords:** *Cucumber green mottle mosaic virus* (CGMMV), iTRAQ, proteomic analysis, watermelon fruit, correlation analysis

## Abstract

*Cucumber green mottle mosaic virus* (CGMMV) is an important viral pathogen on cucurbit plants worldwide, which can cause severe fruit decay symptoms on infected watermelon (usually called “watermelon blood flesh”). However, the molecular mechanism of this disease has not been well understood. In this study, we employed the isobaric tags for relative and absolute quantitation (iTRAQ) technique to analyze the proteomic profiles of watermelon fruits in response to CGMMV infection. A total of 595 differentially accumulated proteins (DAPs) were identified, of which 404 were upregulated and 191 were downregulated. Functional annotation analysis showed that these DAPs were mainly involved in photosynthesis, carbohydrate metabolism, secondary metabolite biosynthesis, plant–pathogen interaction, and protein synthesis and turnover. The accumulation levels of several proteins related to chlorophyll metabolism, pyruvate metabolism, TCA cycle, heat shock proteins, thioredoxins, ribosomal proteins, translation initiation factors, and elongation factors were strongly affected by CGMMV infection. Furthermore, a correlation analysis was performed between CGMMV-responsive proteome and transcriptome data of watermelon fruits obtained in our previous study, which could contribute to comprehensively elucidating the molecular mechanism of “watermelon blood flesh”. To confirm the iTRAQ-based proteome data, the corresponding transcripts of ten DAPs were validated by determining their abundance via quantitative reverse transcriptase-polymerase chain reaction (qRT-PCR). These results could provide a scientific basis for in-depth understanding of the pathogenic mechanisms underlying CGMMV-induced “watermelon blood flesh”, and lay the foundation for further functional exploration and verification of related genes and proteins.

## 1. Introduction

During the infection–defense interaction between viruses and plants, host symptoms are the result of huge intricate disturbance and change of cellular processes [1,2,3,4]. A few years ago, pathogenesis studies were very rare on how virus infection causes pathological changes in plants based on differential expression analysis of protein level. However, in recent years, the rapid development of bioinformatics and proteomics techniques has resulted in a continuously increasing number of studies on quantitative proteomics to elucidate virus–host interactions [5,6,7,8]. Currently, significant advances in high-throughput sequencing (HTS) for RNA profiling to study the mechanisms of pathogenesis have become a major hotspot in life sciences [9,10,11]. However, there are still limitations for transcriptome data to study host–pathogen interactions, as they cannot comprehensively analyze the complex regulatory mechanisms inside host cells, such as post-transcriptional modification, protein expression, protein turnover, degradation, and subcellular localization [5,12,13]. Isobaric tags for relative and absolute quantitation (iTRAQ) technology is a quantitative proteomics technique using isotope labeling that was established in 2004 [14]. This technique has been widely applied in proteome sequencing studies with high accuracy and reproducibility in recent years. Moreover, there have been many reports on the utilization of the iTRAQ technique to study changes in plant physiological metabolisms in response to abiotic stresses, such as cold [15,16,17], heat [18], and salt [19], and biotic stresses, including fungi [20,21], bacteria [22,23], and viruses [24,25].

*Cucumber green mottle mosaic virus* (CGMMV), a member of the genus *Tobamovirus*, is an important global quarantine pathogen infecting the family *Cucurbitaceae* [26]. CGMMV infection usually results in severe disease symptoms on watermelon plants, and especially, it causes a fruit decay called “watermelon blood flesh”. On CGMMV-infected watermelon plants, the inner pulp of fruit transforms to water-soaked dirty red, and the fruit eventually becomes acidified and corrupted [26,27,28]. Using the iTRAQ technique, 38 differentially accumulated proteins (DAPs) were identified from cucumber leaves responding to CGMMV infection [29]. Annotation analysis found that these DAPs included cytochrome complex subunit, phenylalanine-related enzymes, peroxidases, actin, histone, and other protein classes, which mainly participated in host phenylalanine metabolism, phenylpropanoid biosynthesis, and methane metabolic pathways [29]. In a previous study, we have employed high-throughput sequencing technology to analyze transcriptome differences between CGMMV-inoculated and mock-inoculated watermelon fruits [30]. In this study, we further employed iTRAQ combined with high-performance liquid chromatography tandem-mass spectrometry (HPLC-MS/MS) to explore DAPs in watermelon fruits after CGMMV infection.

This study will provide a reference for a comprehensive analysis of the physiological and biochemical mechanisms of “watermelon blood flesh” caused by CGMMV infection. To the best of our knowledge, this is the first report on the proteomic profiles of watermelon fruits in response to CGMMV infection by the iTRAQ technique.

## 2. Results

### 2.1. Symptom Observation and Virus Detection

**Figure 1:**
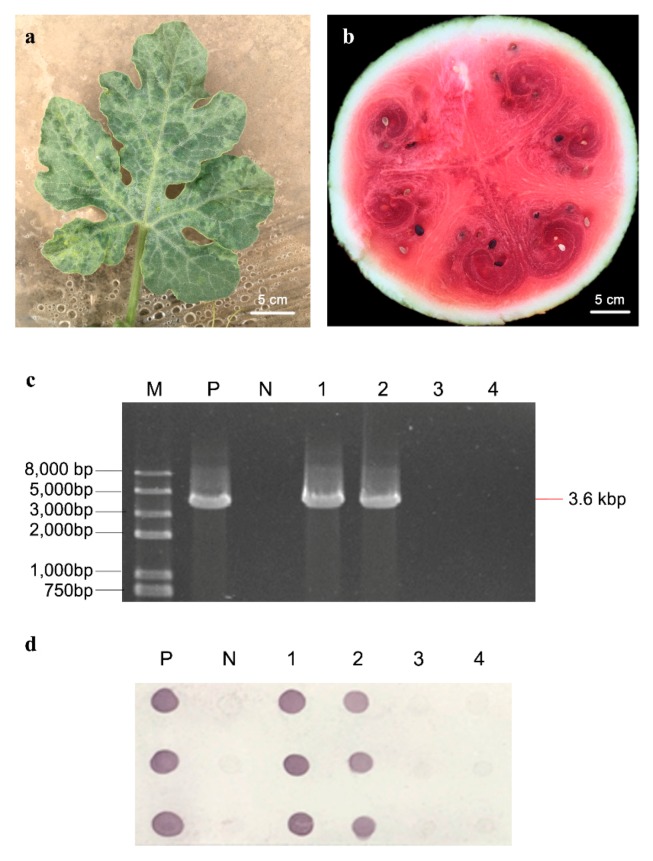
Symptoms and viral detection of *Cucumber green mottle mosaic virus* (CGMMV)-inoculated watermelon plants. (**a**) Symptom of CGMMV-inoculated watermelon leaf, (**b**) symptom of CGMMV-inoculated watermelon fruit, (**c**) reverse transcriptase-polymerase chain reaction (RT-PCR) detection results of inoculated fruit samples with CGMMV-specific primers. M: Trans2K Plus II DNA Marker; P: positive control; N: negative control; lane 1–2: CGMMV-inoculated fruit samples; lane 3–4: mock-inoculated fruit samples. (**d**) Dot enzyme-linked immunosorbent assay (Dot-ELISA) detection results of fruit samples with CGMMV coat protein-specific monoclonal antibody. The sample infected by CGMMV presented a dark brown halo on the nitrocellulose filter membrane.

Watermelon seedlings at 4–6 leaf stage were inoculated with CGMMV (“CGMMV” group) or virus-free phosphate buffer solution (PBS, pH 7.2, “mock” group). The CGMMV-inoculated group exhibited typical foliar mosaic mottling symptoms on leaves at two weeks after inoculation (Figure 1a), and severe fruit decay symptoms, including the internal flesh symptoms of sponginess, rotting, and dirty red discoloration, in watermelon fruits at two months after inoculation (Figure 1b). CGMMV infection of flesh from inoculated plants at two months after inoculation was confirmed by reverse transcriptase-polymerase chain reaction (RT-PCR) and dot enzyme-linked immunosorbent assay (Dot-ELISA) using CGMMV-specific primers and CGMMV coat protein monoclonal antibody (Figure 1c,d). The proteins of flesh sampled from “mock” and “CGMMV” groups (two replicates for each group) at two months after inoculation were extracted and analyzed by the iTRAQ technique.

### 2.2. Identification of Proteins in Response to CGMMV Infection

**Figure 2:**
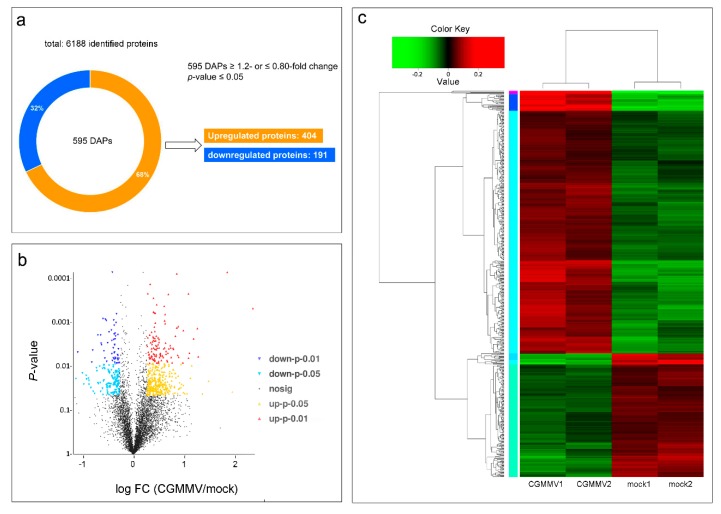
CGMMV-induced differentially accumulated proteins (DAPs) in watermelon fruits. (**a**) Overview of total identified DAPs, (**b**) volcano diagram of the distribution of DAPs. More upregulated proteins (red and yellow dots) were found than downregulated proteins (blue dots). (**c**) Hierarchical cluster of DAPs. CGMMV1 and CGMMV2 were two biological repeats as well as mock1 and mock2. The color from green to red represents protein accumulation level from low to high.

To investigate the effects of CGMMV infection on watermelon fruits at the molecular level, iTRAQ-based quantitative proteomics analysis combined with HPLC-MS/MS was performed to carry out a comparative proteomic analysis between the CGMMV-inoculated and mock-inoculated watermelon fruits. A total of 305,856 spectrograms were identified from four samples (two repeats for “mock” and “CGMMV” groups) by searching the watermelon_v1.fasta database, of which 88,681 spectrograms were known. Among these 37,489 peptides, a total of 6188 proteins in 5149 protein groups were identified at 95% confidence levels (Tables S1 and S2). The data of raw proteins were deposited in the iProX database (http://www.iprox.org) with the accession number IPX0001096001. Among these identified proteins, 595 proteins showed significant changes in their accumulation levels after CGMMV infection based on the criteria of fold change (FC) ≥ 1.20 or ≤0.80, *p*-value ≤ 0.05. The results also revealed that 404 proteins (67.9%) were upregulated, and 191 proteins (32.1%) were downregulated after CGMMV infection (Figure 2a; Supplementary Table S3). Hierarchical clustering and volcano figure of DAPs were obtained (Figure 2b,c). These DAPs might be closely associated with the changes of physiological processes of watermelon fruits infected with CGMMV.

### 2.3. Functional Annotation of DAPs

**Figure 3:**
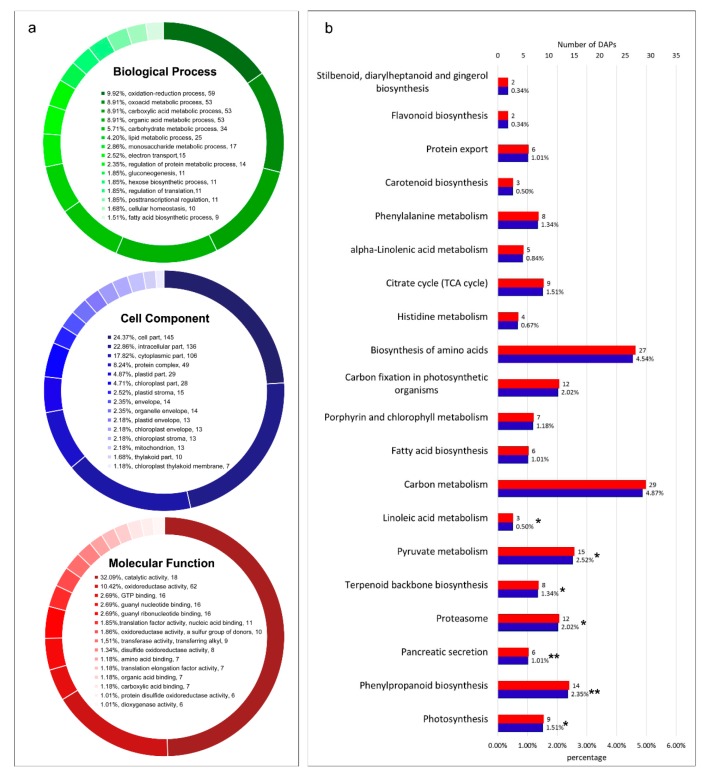
Gene Ontology (GO) function classification and Kyoto Encyclopedia of Genes and Genome (KEGG) pathway enrichment analysis of DAPs. (**a**) GO function classification of DAPs. The three pie charts showed the functional annotation of DAPs in three root categories (“biological process”, “cellular component”, and “molecular function”) of the GO database, respectively. (**b**) KEGG pathway enrichment analysis of DAPs. Top 20 pathways with the smallest *p*-value are demonstrated. Asterisks indicate the significance of enriched pathways (** *p* < 0.01, * *p* < 0.05).

To explore the biological functions of DAPs of watermelon fruits in response to CGMMV infection, we used NCBI non-redundant protein sequences (Nr), Gene Ontology (GO), and Kyoto Encyclopedia of Genes and Genome (KEGG) databases for function annotation analyses (Tables S3–S5). GO annotation analysis showed that these DAPs could be classified into 48 GO terms which belonged to three main categories: “biological processes”, “cellular components”, and “molecular functions” (Figure 3a; Supplementary Table S4). The results of GO enrichment analysis by Goatools software showed that these DAPs mainly participate in metabolic processes, including oxidation-reduction process, carboxylic acid metabolic process, oxoacid metabolic process, carbohydrate metabolic process, lipid metabolic process, electron transport, regulation of protein metabolic process, and regulation of translation. The functions of DAPs were mainly involved in oxidoreductase activity, amino acid binding, and translation elongation factor activity. Moreover, these DAPs were mainly located in plastid stroma, chloroplast part, thylakoid part, and mitochondrion. Meanwhile, based on the KEGG database, these DAPs were allocated to 180 metabolic pathways (Table S5). KEGG pathway enrichment analysis showed that these DAPs were mostly involved in photosynthesis and carbon fixation (photosynthesis, ko00195; carbon metabolism, ko01200; carbon fixation in photosynthetic organisms, ko00710), carbohydrate metabolism (pyruvate metabolism, ko00620; glycolysis/gluconeogenesis, ko00010; starch and sucrose metabolism, ko00500; citrate cycle (TCA cycle), ko00020; amino sugar and nucleotide sugar metabolism, ko00520; galactose metabolism, ko00052), plant defense responses (phenylpropanoid biosynthesis, ko00940; oxidative phosphorylation, ko00190; terpenoid backbone biosynthesis, ko00900; phenylalanine metabolism, ko00360), and protein synthesis and transport (protein processing in endoplasmic reticulum, ko04141; ribosome, ko03010; proteasome, ko03050; glycine, serine, and threonine metabolism, ko00260). The enrichment result of the top 20 KEGG pathways was obtained (Figure 3b).

According to the functional annotation of GO and the KEGG pathway, the DAPs identified in this study were principally involved in photosynthesis, carbohydrate metabolism, secondary metabolite biosynthesis, plant–pathogen interaction, and protein synthesis and turnover. Functional analysis showed that the changes in physiology and biochemistry of watermelon fruits responding to CGMMV infection could be intimately associated with these metabolic processes.

### 2.4. DAPs Involved in Photosynthesis

In our study, 31 DAPs identified from proteomic profiles were associated with photosynthesis (Table S6). Among these, nine DAPs were directly related to photosynthesis, such as ferredoxin, ATP synthase and plastocyanin, and seven DAPs were associated with porphyrin and chlorophyll metabolism, such as magnesium protoporphyrin IX methyltransferase (CHLM), magnesium-protoporphyrin IX monomethyl ester (MPE), and red chlorophyll catabolite reductase (RCCR). In addition, three DAPs, such as 9-cis-epoxycarotenoid dioxygenase (NCED3) and phytoene synthase (Psy), were associated with carotenoid biosynthesis. Moreover, 12 DAPs were associated with carbon fixation in photosynthetic organisms, including four phosphoenolpyruvate carboxykinase (PEPCK). Among DAPs involved in photosynthesis, ATP synthase, RCCR and Psy significantly accumulated to higher levels after CGMMV infection. This was related to preventing production of reactive oxygen species (ROS), reaction of chlorophyll catabolism, and fruit ripening, respectively. The accumulations of NCED3 and most of PEPCKs obviously decreased. This was involved in stress tolerance and provision of CO_2_ for photosynthesis, respectively.

### 2.5. DAPs Involved in Carbohydrate Metabolism

In this study, 58 DAPs related to carbohydrate metabolism were identified in watermelon fruits after CGMMV infection (Table S6). Seventeen DAPs were relevant to pyruvate metabolism (ko00620). Therein, the expression levels of one phosphoenolpyruvate carboxylase (PEPC), one malate dehydrogenase (MDH), and one NADP-dependent D-sorbitol-6-phosphate dehydrogenase (NADP-S6PDH) were induced, whereas three pyruvate kinase (PK) and five phosphoenolpyruvate carboxykinase (PEPCK) were significantly downregulated after CGMMV infection. Thirteen DAPs were correlated to glycolysis/gluconeogenesis (ko00010), of which two fructose-bisphosphate aldolase (FBA) and one NADP-dependent glyceraldehyde-3-phosphate dehydrogenase (NADP-GAPA) were upregulated, whereas one hexokinase-3 (HK3) and one leghemoglobin reductase (FLbR) exhibited significantly decreased levels of accumulation in CGMMV-inoculated watermelon fruits. Twelve DAPs were associated with starch and sucrose metabolism (ko00500), among which one UDP-glucose 6-dehydrogenase 1 (UGD1), two UDP-glucuronic acid decarboxylase 1 (UXS1), and two beta-glucosidase (GLU12) were upregulated, whereas one lysosomal beta glucosidase (gluA) and one beta-glucosidase 40 (GLU40) distinctly exhibited decreased accumulation. Moreover, several key enzymes or proteins involved in citrate cycle (ko00020) and galactose metabolism (ko00052) were differentially accumulated in CGMMV-inoculated watermelon fruits.

### 2.6. DAPs Involved in Secondary Metabolites Biosynthesis

The results showed that 53 proteins involved in secondary metabolites biosynthesis exhibited a significantly fluctuant level of accumulation after CGMMV infection (Table S6). These DAPs belonged to phenylpropanoid biosynthesis (ko00940), phenylalanine metabolism (ko00360), terpenoid backbone biosynthesis (ko00900), fatty acid metabolism (ko01212), fatty acid biosynthesis (ko00061), alpha-Linolenic acid metabolism (ko00592), linoleic acid metabolism (ko00591), and flavonoid biosynthesis (ko00941). These results showed that the secondary metabolites biosynthesis was seriously affected by CGMMV infection in watermelon fruits, and most secondary metabolites were especially associated with defense pathways.

### 2.7. DAPs Involved in Plant–Pathogen Interaction

Plants respond to pathogen attacks by a rapid change in gene expression level, which leads to the different accumulation of pathogenesis-related proteins. In this study, 19 DAPs were identified to participate in the plant–pathogen interaction pathway (Table S6). Among these, eight DAPs were upregulated at the protein level, including five thioredoxins (TRX), two endoglucanases, and one ent-kaurene oxidase. Meanwhile, the expression levels of 11 DAPs were decreased, including four heat shock proteins (HSPs), one secologanin synthase (SLS), and one sugar transporter (ERD6). These DAPs might be specific for “watermelon blood flesh” caused by CGMMV infection.

### 2.8. DAPs Involved in Protein Synthesis and Turnover

The replication, assembly, proliferation, and movement of plant viruses require many host proteins, and affect the protein synthesis and turnover of the host. In this study, 101 DAPs involved in the protein synthesis and turnover were obtained (Table S6). Among them, 14 DAPs belonged to protein processing in endoplasmic reticulum (ko04141), including protein transport protein Sec24, GTP-binding protein SAR1A, and endoplasmic reticulum oxidoreductin-1. Interestingly, 14 DAPs involved in ribosome (ko03010, such as 40S ribosomal protein S14-2 and 60S ribosomal protein L22-2), and six DAPs involved in protein export (ko03060, such as signal recognition particle proteins), showed upregulation at the protein levels. In addition, the accumulation of several translation initiation factor-related proteins, E3 ubiquitin protein ligase-related proteins, nucleoside diphosphate kinase, and cysteine-rich receptor-like protein kinase, which related to translation initiation, ubiquitin-mediated pathway, and kinase phosphorylation, were significantly affected by CGMMV infection.

### 2.9. Correlation Analysis of Proteome and Transcriptome Data

In a previous study, we had analyzed the transcriptomic changes of watermelon fruits inoculated with CGMMV via RNA-Sequencing (RNA-Seq) technology [26]. To comprehensively clarify the molecular mechanism underlying fruit decay caused by CGMMV infection, a correlation analysis of multiple omics was further conducted by integrating these two sets of data (RNA-Seq and iTRAQ) in this study. The results showed that a total of 253 annotated genes and their proteins showed differential expression in infected watermelon fruits, of which 107 showed upregulation at gene and protein levels, 58 showed downregulation at both levels, 21 showed upregulation at the gene level while downregulation at the protein level, and 67 showed downregulation at the gene level while upregulation at the protein level. Totally, 165 of 253 pairs showed consistent regulation trends after CGMMV infection (Table S7).

**Figure 4:**
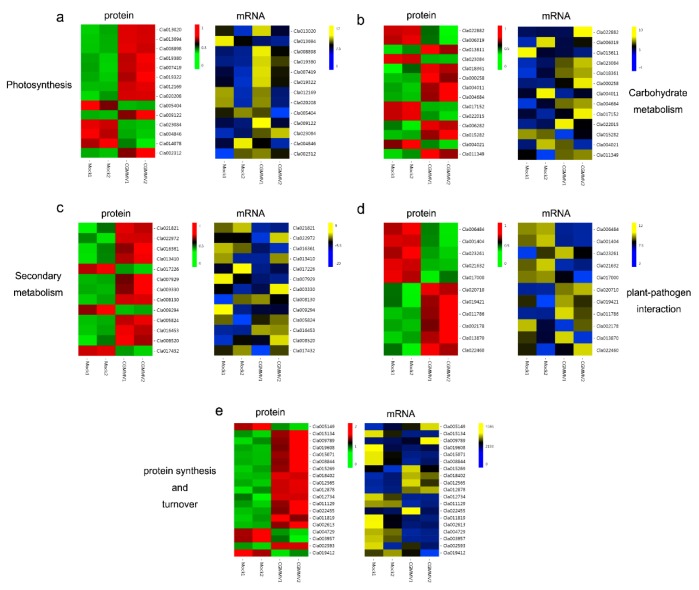
Correlation analysis of DAPs and their corresponding genes involved in main metabolic processes. The color (from green to red at the protein level; blue to yellow at the transcriptional level) represents protein accumulation level or gene expression level from low to high. (**a**) Photosynthesis, (**b**) carbohydrate metabolism, (**c**) secondary metabolites biosynthesis, (**d**) plant–pathogen interaction, and (**e**) protein synthesis and turnover.

The main biological functions of DAPs identified in this study were analyzed (Table S6). A further correlation analysis was performed on 72 DAPs involved in these major metabolic pathways (Figure 4; Table S8). Among them, 14 pairs were related to photosynthesis, of which ten were upregulated and four downregulated at the proteomic level, and nine were upregulated and five downregulated at the transcriptional level. Nine pairs, which were related to ferredoxin-like, ATP synthase, plastocyanin, CHLM, NCED3, Psy, and PEPCK, showed consistent expression trends. As for the carbohydrate metabolism pathway, five of 15 pairs mainly associated with HK, PEPCK, PK, UGD1, UXS1, OGDH, and UGE showed the similar accumulation trends in response to CGMMV infection. Eight carbohydrate metabolism-related proteins and nine genes appeared to be upregulated. Most of the proteins and genes related to phenylpropanoid, phenylalanine, terpenoid backbone, fatty acid, linoleic acid, and flavonoid biosynthesis or metabolism exhibited consistent upregulation or downregulation. Nine of eleven pairs involved in the plant–pathogen interaction showed similar expression patterns, such as heat shock protein and thioredoxin. In the protein synthesis and turnover pathway, nine of 19 pairs showed the same expression trends. Interestingly, the majority of the ribosomal proteins and elongation factors had opposite expression trends at the transcriptional and proteomic levels.

### 2.10. Validation of iTRAQ Data by qRT-PCR

**Figure 5:**
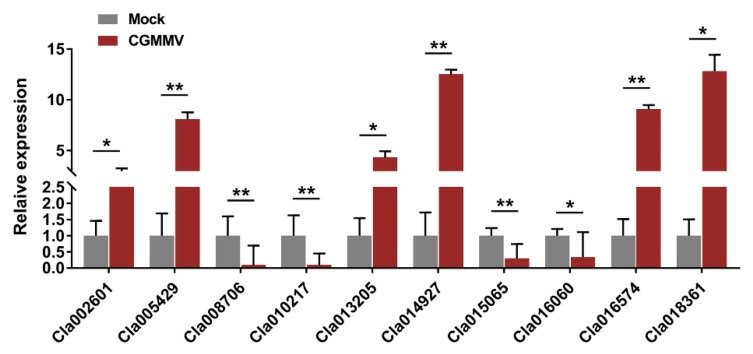
Validation of isobaric tags for relative and absolute quantitation (iTRAQ) data by quantitative reverse-transcription PCR (qRT-PCR). The watermelon 18S rRNA gene was used as an internal control. Three independent experiments were conducted with at least three biological replicates each. Error bars represent mean ± standard deviation (SD). Asterisks indicate statistically significant differences compared to the control (Student’s *t*-test). (** *p* < 0.01, * *p* < 0.05).

To validate the data from iTRAQ-based proteomic profiles, we randomly selected ten DAPs to determine their relative transcript abundance by quantitative reverse-transcription PCR (qRT-PCR). These DAPs included proteins that were associated with carbohydrate metabolism, such as pectinase (Cla014927) and pyruvate kinase (Cla018361), proteins associated with photosynthesis, such as oxygen-evolving enhancer protein (Cla005429), and proteins associated with stress responses, such as heat shock proteins (Cla016060) and stress proteins (Cla015065). These ten genes exhibited significantly differential expression in watermelon fruits after CGMMV infection (Figure 5), and their expression trends were consistent with the changes in abundance of the corresponding proteins, as revealed by the iTRAQ technique (Table S9).

## 3. Discussion

CGMMV, belonging to the genus *Tobamovirus*, is one of the most widely occurring and damaging viruses on watermelon plants [31]; however, fruit decay and acidification symptoms are only observed on CGMMV-infected watermelons. China is the largest watermelon producing country worldwide. It produced approximately 63 million tons of watermelon, accounting for almost 60.62% of the total global watermelon production in 2018 (http://faostat.fao.org/). Considering the potential threat to the production of cucurbit crops, CGMMV has been listed as a quarantine pest by the Chinese government in May 2007 [32]. The miRNA and transcriptome profiles of watermelon leaves responding to CGMMV infection have been studied in recent years [33,34]. However, there are still no reports on proteomic profiles in response to CGMMV infection in watermelon, especially in fruits. In a previous study, we utilized HTS technology to analyze the transcriptomic changes of CGMMV-inoculated watermelon fruits [30]. In this study, we further employed the iTRAQ technique to carry out a comparative study of the proteomic profiles between CGMMV-inoculated and mock-inoculated watermelon fruits to explore the molecular mechanisms underlying “watermelon blood flesh” caused by CGMMV infection. The proteomics data presented in this study provided novel insights into the responses of watermelon fruits to CGMMV infection and a scientific basis for the better understanding of the molecular mechanism of “watermelon blood flesh”.

### 3.1. Changes in Photosynthesis after CGMMV Infection

Photosynthesis, as a special and basic life process of green plants, is the main factor that determines the yield and quality of products. However, chlorophyll content, maximal quantum yield of photosynthesis, and photosynthetic rate of plants changed after virus infection, which suggests that host photosynthesis is affected by the process of virus infection [35]. A decreased photosynthesis efficiency was found upon CGMMV infection in the early stages of watermelon leaves [33]. In this study, 31 DAPs were associated with photosynthesis, including ATP synthase, RCCR, Psy, NCED3, and PEPCK (Tables S6 and S8; Figure 4a). Chloroplastic ATP synthase builds up a proton motive force preventing production of reactive oxygen species in photosystem I [36]. RCCR, belonging to the chlorophyll catabolic enzymes (CCEs), catalyzes the key reaction of chlorophyll catabolism, porphyrin macrocycle of pheophorbide (Pheide), to a primary fluorescent catabolite (pFCC) [37]. Psy plays a key role in carotenoid biosynthesis, which is enhanced during fruit ripening [38]. In our study, the accumulations of these proteins were significantly upregulated in CGMMV-inoculated watermelon fruits compared with that in mock-inoculated watermelon fruits. These results suggested that an acceleration of chlorophyll catabolism was induced by CGMMV infection, and the infected fruits were overripe with more reactive oxygen. PEPCK functions in the provision of CO_2_ for photosynthesis [39]. In our study, most of PEPCKs exhibited an obviously decreased accumulation, which might reduce the provision of CO_2_ and inhibit carbon fixation for photosynthesis. Ferredoxin is the central hub connecting photosystem I to cellular metabolism, and plastocyanin participates in electron transfer between P700 and the cytochrome b6-f complex in photosystem I [40]. CHLM is essential for the formation of chlorophyll and subsequently for the formation of photosystems I and II and cytochrome b6f complexes [41]. These proteins are important components of the photosynthetic system, which were significantly affected by CGMMV infection in this study. These differential accumulations of photosynthetic proteins might be associated with photosynthetic efficiency and the rate of carbon fixation, which indirectly affected the organic matter content in watermelon fruits.

### 3.2. Changes in Carbohydrate Metabolism after CGMMV Infection

The products of carbohydrate metabolism are important substances involved in the growth and development of plants. Virus infection can change host carbohydrate metabolism and directly affect fruit quality [42]. In this study, 58 DAPs were identified to be related to carbohydrate metabolism, including PK, HK3, CS, UGE, and PEPCK (Tables S6 and S8; Figure 4b). PK is one of the major rate-limiting enzymes in the glycolysis pathway that catalyzes the essentially irreversible transfer of Pi from phosphoenolpyruvate to ADP, yielding pyruvate and ATP. The cytosolic pyruvate kinase 1 gene is induced specifically during the resistance response to *Tobacco mosaic virus* (TMV) in *Capsicum annuum* [43]. Mitochondria-associated HKs play a critical role in the control of programmed cell death (PCD) in plants, and their higher levels are supposed to improve the resistance to oxidative stress and pathogen infection [44]. As CS catalyzes the first committed step, it has a key role in the TCA cycle of the mitochondria of all organisms, especially in plants, since mitochondrial activities have to be coordinated with photosynthesis [45]. UGE may be involved in the regulation of cell wall carbohydrate biosynthesis [46]. In our study, we found that the expression levels of CS and UGE were induced, while PK and HK3 were significantly downregulated after CGMMV infection. Significant changes in sugar contents (sucrose, glucose, and fructose) of watermelon fruits were found after CGMMV infection [47], and the genes related to pyruvate metabolism and the fermentation pathway were also strongly affected by CGMMV infection at the transcriptional level [30]. Therefore, it is speculated that CGMMV infection may influence the pyruvate metabolism and citrate cycle, and further cause a disturbance in the energy supply and sugar content in the watermelon fruits. Moreover, the resistance to oxidative stress and pathogens may be reduced in infected watermelon fruits, and the cell wall carbohydrate biosynthesis can be influenced by CGMMV infection, which will exacerbate the degree of “watermelon blood flesh” disease.

### 3.3. Changes in Secondary Metabolites Biosynthesis and the Plant–Pathogen Interaction Pathway after CGMMV Infection

Secondary metabolite biosynthesis plays an important role not only in growth and development of plants, but also in resistance to stresses, and the metabolites can function in defense-related signaling processes [48]. The ubiquitously distributed peroxiredoxins (Prxs), such as 2-Cys Prx, atypical 2-Cys Prx, and 1-Cys Prx, have been shown to function in diverse cellular defense-signaling pathways, especially intracellular ROS activate defense signaling pathways [42]. In addition, OPR, a key enzyme in the biosynthesis of jasmonic acid (JA), also plays an important role in plant defense responses [49]. JA is essential for systemic resistance against plant viruses, such as TMV [50]. In this study, the accumulation levels of 1-Cys Prx and OPR2 were upregulated after CGMMV infection, indicating that watermelon plants might turn on a series of secondary metabolism processes in resistance to CGMMV infection. Moreover, HSP70s are involved in microbial pathogenesis, cell death responses, and immune responses. Several RNA virus infections, such as TMV, CMV, and WMV, induce HSP70 expression and HSP70s regulates virus infection in turn [51]. TRX enzymes play important roles in diverse aspects of plant immune signaling [52]. In this study, five TRX proteins were upregulated and four HSPs were downregulated in the plant–pathogen interaction pathway (Tables S6 and S8; Figure 4c,d), indicating that these DAPs might play vital roles in watermelon immunity against CGMMV infection.

### 3.4. Changes in Protein Synthesis and Turnover after CGMMV Infection

Replication and intercellular movement of plant viruses depend on host mechanisms supporting the formation, transport, and turnover of functional complexes between viral genomes, virus-encoded products, and cellular factors [53]. This complex process of plant virus infection requires participation and cooperation of host proteins, which activates the biological processes of host protein synthesis and turnover. In this study, we found that all the DAPs in ribosome (ko03010) and protein export (ko03060), and most of the DAPs in biosynthesis of amino acids (ko01230) were upregulated in CGMMV-inoculated watermelon fruits, suggesting the involvement of host protein synthesis and turnover in the process of CGMMV infection. Catalase and NDPK have essential roles in ROS signaling, and a strong interaction between NDPK2 and CAT1 possibly plays a vital role in the antioxidant defense against ROS [54]. The cysteine-rich RLKs (CRKs) represent a prominent subfamily of transmembrane-anchored receptor-like protein kinases (RLKs) related to the physiological process of stress responses. Transient silencing of barley (*Hordeum vulgare*) CRK gene *HvCRK1* expression led to enhanced resistance to the pathogen *Blumeria graminis* f.sp. *hordei* (*Bgh*) [55]. In this study, among DAPs related to kinase phosphorylation, NDPK2 was upregulated, and CRK10 was downregulated (Tables S6 and S8; Figure 4e), suggesting that watermelon might turn on defense strategies to counter CGMMV infection. It is probably the case that the system of host mechanism not only endures the synthesis of many proteins for virus replication and movement, but also activates the synthesis and turnover of massive resistance proteins in response to CGMMV infection.

### 3.5. Correlation Analysis of Proteome and Transcriptome Data

In a previous study, we identified 1621 differentially expressed genes (DEGs) from the transcriptome profiles of CGMMV-inoculated and mock-inoculated watermelon fruits [30]. Since the expressions of many genes are regulated at the translational levels, proteome analysis is required for deeply understanding the defense mechanism. In this study, 595 DAPs were identified and then correlated with the DEGs obtained from the previous study by the same accession number in the watermelon genomics database (http://cucurbitgenomics.org/). Correlation analysis results showed that the expressions of 42.5% DAPs (253/595) were associated with that of the DEGs, and 165 of 253 pairs showed consistent expression trends.

Due to the limitations in the sensitivity of identification methods of present proteins, changes in abundance of some proteins were usually not detected [13,24]. Additionally, discrepancies between transcript and protein levels might originate from specific biological metabolic processes. Protein abundance reflects a dynamic balance among a series of biological processes during protein synthesis and maintenance in cells. These biological processes include transcription, mRNA processing and degradation, translation, localization, and protein modification and processing [13]. Post-transcriptional regulatory mechanisms can be important biological reasons for the low correlation between transcriptome and proteome data. The mRNAs can still be detected when translation is inhibited, while they cannot be translated into corresponding proteins [56,57]. Our study found that CGMMV infection significantly affected the accumulation levels of many ribosomal proteins and translation initiation factors in watermelon fruits (Table S6), which played important roles in post-transcriptional regulatory mechanisms [24]. These ribosomal proteins could stabilize the formation of ribosome around the start codons as well as affect translation efficiency. Additionally, translation elongation factors could regulate the initial stages of protein synthesis [58]. The differential expressions of ribosomal proteins and translation initiation factors could influence the consistence of abundance of proteins and mRNAs.

**Figure 6:**
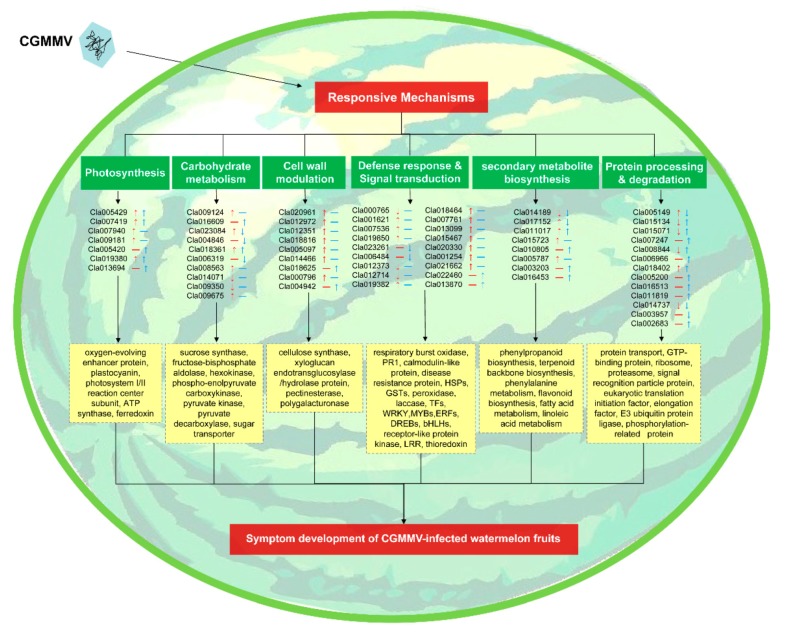
Potential regulation network of a CGMMV-responsive mechanism in watermelon fruits. The arrows and dashes following the gene ID represent the expression patterns of DEGs (red) and DAPs (blue).

To further comprehensively elucidate the molecular mechanism underlying fruit decay caused by CGMMV infection, we plotted a hypothetical regulation network map of a potential CGMMV-responsive mechanism in watermelon fruits (Figure 6). Correlation analysis results showed that CGMMV infection could strongly affect the photosynthesis, carbohydrate metabolism, cell wall modulation, defense response and signal transduction, secondary metabolites biosynthesis, and protein processing and degradation in watermelon fruits. The symptoms of CGMMV-infected watermelon fruits, such as pulp deterioration and acidification, might be results of these metabolic disorders. 

In summary, integration analysis of proteomics and transcriptomes in response to CGMMV infection in watermelon fruits can help for in-depth understanding of various physiological and biochemical mechanisms, and provide a scientific basis to explore the formation of “watermelon blood flesh”, and lay the foundation for further functional exploration and verification of related genes and proteins. However, it is limited by the depth of current omics analysis techniques and our understanding of virus–host interactions. More in-depth molecular biological research is necessary to fully clarify the mechanism of CGMMV infection in watermelon fruits.

## 4. Materials and Methods

### 4.1. Virus Inoculation and Sample Collection

Watermelon plants (*Citrullus lanatus* cv “Jingxin No.3”) were utilized for this study. The CGMMV virus source (isolate: CGMMV-lnxg; GenBank ID: KY040049) was preserved on bottle gourd plants (*Lagenaria siceraria* (Molina) Standl.) by the Plant Virus Laboratory, College of Plant Protection, Shenyang Agricultural University, China. The plant seeds produced by Beijing Vegetable Research Center (BVRC) were bought from local seed stores. Gourd leaves containing CGMMV were added at a 1:10 ratio (*m*/*v*) into 0.01 mol·L^−1^ PBS (pH 7.2) and fully ground and homogenized before use. The treatment group (“CGMMV”) was mechanically inoculated with CGMMV while the control group (“mock”) was inoculated with PBS buffer only at 4–6 leaf stage. One hundred plants were used in each treatment. One week later, the watermelon seedlings were grafted onto virus-free squash rootstocks and transferred to a greenhouse at a melon plantation in Shenyang, Liaoning Province, China (latitude: 41.9511031186; longitude: 122.7212245701). The greenhouse was covered with polyethylene (PE) film with good light transmittance, the photoperiod was 10 to 12 h of sunshine, the temperature was kept at 28 to 32 °C in the daytime and 18 to 20 °C at night with proper ventilation and cooling, and the air humidity was kept at 80% to 90%. Water was supplied with a drip irrigation system and a potassium sulfate compound was used as fertilizer. Artificial pollination, pruning, and fruit setting were performed according to routine field management protocols. Fruits samples from these two groups were separately collected at two months after inoculation. The central flesh was obtained for the virus detection via RT-PCR (forward primer: 5′-GTTTTAATTTTTATAATTAAACAAACAAC-3′; reverse primer: 5′-GTTCTGCATTAATTGCTATTTGG-3′) and Dot-ELISA (with CGMMV coat protein-specific monoclonal antibody). The central flesh of at least ten fruits per group was mixed as one sample and immediately frozen in liquid nitrogen for iTRAQ analysis. For each group, two biological repeats were performed in this study. 

### 4.2. Protein Extraction and iTRAQ Analysis

The collected watermelon flesh samples were ground in liquid nitrogen and then homogenized with lysis buffer (7 M urea and 4% sodium dodecyl sulfate), followed by 40 s ultrasonication. Then, the debris and broken cells were removed through centrifugation at 25,000× *g* at 4 °C for 15 min. Proteins were extracted using the trichloroacetic acid (TCA)-acetone method and the protein concentration of each sample was estimated with the bicinchoninic acid assay (BCA). Subsequently, the obtained proteins were digested by trypsin (6 ng/μL in 50 mM ammonium bicarbonate solution) at 37 °C for 12 h. Then, the digested products were labeled with iTRAQ reagent according to the manufacturer’s instructions. The “CGMMV” groups were labeled as 113 and 114, while “mock” groups as 115 and 116.

### 4.3. RPLC First-Dimensional Separation 

Equal labeled samples were pooled, and peptides were separated using a reverse-phase chromatography column on a Waters ACQUITY UPLC system (Waters, Milford, MA, USA). Briefly, each labeled sample was dissolved in 50 μL mobile phase A solution (2% ammonium hydroxide and formic acid in ddH_2_O, pH 10) and then centrifuged at 14,000× *g* at 4 °C for 20 min. The supernatant was loaded onto a column (2.1 mm × 150 mm X Bridge BEH300, Waters) and then eluted stepwise to get collected fractions by injecting mobile B solution (100% acetonitrile). The UV detection wavelength was 214 nm/280 nm, flow rate was 400 μL·min^−1^, and gradient was 60 min. The collected fractions were combined to produce 10 fractions and lyophilized.

### 4.4. LC-MS/MS Analysis

The obtained fractions were separated using a C^18^ reverse-phase chromatography column (74 μm × 25 cm; Thermo Fisher Scientific, USA) on an EASY-nLC 1200 chromatography system (Thermo, Waltham, MA, USA). The chromatography separation time was 90 min. Solvent A was 2% ACN (containing 0.1 % formic acid) and solvent B was 80% ACN (containing 0.1% formic acid). The flow rate was 300 μL·min^−1^ and the elution gradient was set as follows: the initial ratio of solvent B was 2%, which was increased to 40% at 70 min, increased to 90% after an additional 6 s, before decreasing to 2% after 75.1 min. The fractionated peptides were analyzed by a Thermo Scientific Q Exactive (Thermo). The parameter settings for the mass spectrometer were as follows: scanning range, 350–1300 *m*/*z*, acquisition modes, DDA and top 20, primary mass spectrometry resolution rate, 70,000 *m*/*z*, fragmentation method, HCD, secondary mass spectrometry resolution rate, 17,500 *m*/*z*, and dynamic discharge time, 18 s. Thermo Xcalibur 4.0 software (Thermo) was used for data acquisition.

### 4.5. Bioinformatics Analysis of Proteins

The raw mass data were processed for the peptide data analysis using Proteome Discoverer 2.1 (Thermo) with a false discovery rate (FDR) ≤ 0.01 for searching the watermelon_v1.fasta protein database (http://cucurbitgenomics.org/). According to the protein abundance, DAPs were identified by using a cutoff FC value between the treatment and control groups based on the criteria of fold change (FC) ≥ 1.20 or ≤ 0.80, *p*-value ≤ 0.05. Molecular functions of the identified DAPs were annotated by Nr, GO, and KEGG databases. GO enrichment analysis was performed by Goatools program (https://github.com/tanghaibao/GOatools) and KEGG pathway analysis of DAPs was performed by KOBAS (http://kobas.cbi.pku.edu.cn/home.do).

### 4.6. Correlation Analysis between iTRAQ and RNA-Seq

Comparative analysis of transcriptome profiles of CGMMV-inoculated and mock-inoculated watermelon fruits has been carried out in our previous study [30]. The transcriptome data have been deposited in NCBI Sequence Read Archive (SRA, http://www.ncbi.nlm.nih.gov/Traces/sra) with the accession numbers SRR565820-SRR565824. In order to obtain a “panoramic” spectrum of watermelon fruits in response to CGMMV infection, we carried out a comprehensive analysis of the differential accumulation levels of genes and proteins to achieve the complementation of multiple omics. Moreover, the watermelon fruit samples used for iTRAQ analysis in this work were the same batch of samples that were previously used for RNA-Seq, which ensured the comparability of the data. The proteins and genes were correlated by the same accession number in the watermelon genomics database (http://cucurbitgenomics.org/).

### 4.7. Quantitative Reverse-Transcription PCR

To validate the iTRAQ data, ten DAPs were randomly selected for analysis of their corresponding gene transcript levels by qRT-PCR with specific primers (Table S9). Total RNA was extracted from watermelon fruits using the TRIzol reagent (Invitrogen, Waltham, MA, USA) and qRT-PCR was performed using SYBR Premix Ex Taq II (TaKaRa, Kusatsu, Japan) following the manufacturer’s protocol. qRT-PCR was conducted on an ABI StepOne Real-Time PCR System (Applied Biosystems, Waltham, MA, USA) as follows: 3 min at 95 °C for denaturation, followed by 40 cycles of denatured at 95 °C for 7 s, annealing at 57 °C for 10 s, and extension at 72 °C for 30 s. The watermelon 18S rRNA gene was used as an internal reference gene. The 2^−ΔΔ*C*t^ method was used to analyze the relative expression of genes [59]. All gene expression analyses were performed using three independent biological replicates.

## Supplementary Materials

Supplementary materials can be found at https://www.mdpi.com/1422-0067/21/7/2541/s1.
